# New Damage Evolution Law for Steel–Asphalt Concrete Composite Pavement Considering Wheel Load and Temperature Variation

**DOI:** 10.3390/ma12223723

**Published:** 2019-11-11

**Authors:** Xunqian Xu, Xiao Yang, Wei Huang, Hongliang Xiang, Wei Yang

**Affiliations:** 1 National & Local Joint Engineering Research Center of Technical Fiber Composites for Safety and Health, Nantong University, Nantong 226019, China; xunqian_xu@ntu.edu.cn (X.X.); yangxiao_ntu@163.com (X.Y.); hongliang_xiang@163.com (H.X.); weiyang_ntu@163.com (W.Y.); 2 Intelligent Transportation System Research Center, Southeast University, Nanjing 210096, China

**Keywords:** long-span steel bridges, steel bridge deck pavement (SBDP), epoxy asphalt (EA), fatigue damage evolution law, micro properties, fatigue test

## Abstract

Epoxy asphalt (EA) concrete is widely used in constructing long-span steel bridge pavements (SBDPs). This study aims to derive a fatigue damage evolution law, conducting an experimental investigation of SBDP. First, a general theoretical form of the fatigue damage evolution law of materials is established based on the thermal motion of atoms. Then, fatigue experiments demonstrate that this evolution law well represents the known damage–life relationships of SBDP. Taking into account the experimental relationships between damage and fatigue life under symmetrical cyclic loadings with different overload amplitudes and temperature variations, a detailed damage evolution law is deduced. Finally, the role of damage accumulation is discussed on the basis of the proposed damage evolution law for the extreme situation of heavy overload and severe environments. The results show that both heavy loading and falling temperatures increase the fatigue damage of SBDP considerably. EA shows a fatigue life two to three times longer than that of modified matrix asphalt (SMA) or guss asphalt (GA). For the same thickness, EA pavement is demonstrated to be more suitable for an anti-fatigue design of large-span SBDP under high traffic flows and low temperatures.

## 1. Introduction

A number of large-span steel bridges with orthotropic deck plates have been constructed in the United States, Japan, and China, owing to their relatively light weight, small structural depth, and large load capacity. However, these steel bridge decks were too thin, leading to a large local deformation in the deck pavement; further, fatigue cracks have been recorded in many types of pavement structures within only 1–2 years [1,2,3]. Overloading owing to heavy-duty vehicles is the main reason behind pavement damage. As steel bridge pavements are often exposed to a large variation in temperature, there is an increased demand for paving materials with considerably higher service temperature requirements. Moreover, there is a lack of design methods and theoretical research in this aspect [4,5]. In recent years, many studies have conducted experiments and theoretical analyses on the mechanical properties and the anti-fatigue performance of asphalt pavements of steel bridge decks, aiming to improve their fatigue reliability [6,7,8].

Cracks in bridge deck pavement layers not only degrade their mechanical performance but also induce secondary deterioration, which can greatly shorten the service life of bridges [9]. Di Benedetto et al. investigated the fatigue characteristics of a dense graded asphalt concrete mixture by using 11 different test methods, comprising uniaxial tension/compression and 2-, 3-, and 4-point bending and indirect tension tests [10]. They concluded that the fatigue test results obtained using the classical fatigue approach are considerably influenced by the test type and mode of loading (controlled stress or strain) used [11,12]. In the past 30 years, the fatigue performance of asphalt concrete has been analyzed through different test methods, and large numbers of research outcomes have been established [13,14,15,16,17,18,19]. By comparing these different fatigue assessment methods, Qian et al. concluded that the trabecular bending fatigue test and splitting fatigue test have clear advantages and can be easily applied to the fatigue assessment of asphalt concrete pavement materials and surface layer structures [20]. Campbell combined the two commonly used modes of the fatigue test (constant stress and constant strain) in order to reflect more complex loading conditions for pavement materials [21]. Shen et al. suggested that the strain control method (constant strain mode) can be used to evaluate mixtures for thin asphalt pavements (thickness less than 5.08 cm) because the strain in the asphalt layer is governed by the underlying steel deck and is not affected by a decrease in the stiffness of the pavement itself; in contrast, the stress controlled fatigue test (constant stress mode) is more suitable for asphalt pavements with a larger thickness (thickness greater than 15.24 cm) [22].

Saboo and Kumar predicted the fatigue crack behavior of asphalt concrete based on Miner’s cumulative damage theory, and the relationship between the tensile strain of specimens and the number of circular loading cycles was also studied [23]. Up until now, several approaches have been developed to predict the fatigue life of asphalt pavements including the phenomenological method, the continuous damage mechanics method, the fracture mechanics method, and the dissipated energy method [24,25,26,27]. Common methods to derive the *N_f_* equation include the strain-related fatigue model, the strain- and stiffness-related fatigue model, and the fatigue model combining the strain, stiffness, and volume parameters. These models are simple in form, however, they cannot accurately describe accumulative damage processes under cyclic loading [28,29,30].

Epoxy asphalt (EA) concrete is a type of artificial composite material containing original defects. The fatigue damage and failure of bridge deck pavements are essentially the macro indicators of their microstructure accumulated damage and deformation failure [31]. Researchers have studied the fatigue damage performance of structures and materials and proposed both indirect and direct measurement techniques [32,33,34]. However, internal fatigue damage is often generated in materials and developed under a level of alternating stress far less than the material strength; this mechanism of damage evolution has not been clearly understood [35]. One theory holds that internal defects of materials can lead to the evolution and expansion of damage caused by local stress concentrations of structures and materials. This theory ignores that alternating stress is the main factor causing fatigue and fails to interpret the fatigue mechanism well [36,37]. Owing to the fatigue damage evolution mechanism being unclear, a fatigue life assessment and the critical fatigue damage evolution law often provide some empirical regularity according to the result of a large number of trials. Simulation models not only lack a certain theoretical basis but also are time-consuming and contain undetermined parameters, making them difficult to be employed for engineering applications. It can be said that the key to establishing a damage evolution law with a certain theoretical basis and clear physical significance lies in considering the evolution mechanism of fatigue damage from a microscopic perspective and an analysis of the mechanical properties and fatigue damage behavior from a macroscopic perspective.

In this study, the fatigue damage evolution mechanism of asphalt concrete materials is studied from a microscopic perspective (namely, the thermal motion of atoms), and a general theoretical form of the fatigue damage evolution law of asphalt concrete materials is given. The fatigue lives of several asphalt concretes are assessed. The feasibility and rationality of the proposed method are verified through a comparison between the calculated results and practical engineering cases of EA concrete in long-span steel bridges. At the same time, modified matrix asphalt (SMA) and guss asphalt (GA) are compared and analyzed. The EA concrete can provide better anti-fatigue performance under different loading conditions than the SMA and GA concretes.

## 2. Materials and Methods

### 2.1. Theoretical Model

#### 2.1.1. General Formulation

The minimum active energy for atoms to escape, *Q*, can be expressed in the following forms [38]:

(1) $$Q=\frac{{\left(\tau_{b}-\tau \right)}^{2}}{2G},$$

(2) $$Q=\frac{{\left(\tau_{b}-q\tau \right)}^{2}}{2G},$$

(3) $$Q=\frac{\tau_{b}^{2}-\tau^{2}}{2G},$$

where *τ_b_* is the ideal shear strength, *τ* is the external shear stress, *G* is the shear modulus, and *q* is the concentration coefficient of the stress.

Generally, the probability that an atom has an energy greater than a certain critical active energy *Q* owing to thermal disturbance is [38]:

(4) $$P=\exp\left(-\frac{Q}{kT}\right),$$

where *T* is the thermodynamic temperature, *k* is Boltzmann’s constant, and *kT* = 1/40 eV is the expression of the average kinetic energy of atoms at room temperature.

The rate of creep and damage evolution of materials induced by this escape process can be written as [38]:

(5) $$v=A\exp\left(-\frac{Q}{kT}\right),$$

where *A* is the material coefficient and represents the ratio of the probability of atoms escaping to the rate of creep and damage evolution. Equation (5) is used to determine the rate of strain in the creep phenomenon ($\dot{\varepsilon }=v$). During the process of damage evolution, the rate given by Equation (5) is the rate of damage evolution.

The critical activity of materials under repeated loading stress can be related to the current damage degree and stress as *Q*(*σ_a_*, *σ_m_*, *D*), where *σ_a_*, *σ_m_*, and *D* are the stress value of cyclic load, the average stress value, and the current damage state (damage degree value) of the material, respectively. Using Equation (5), the general form of fatigue damage evolution can be given as [38]:

(6) $$\frac{dD}{dN}=A\exp\left(-\frac{Q\left(\sigma_{a},\sigma_{m},D\right)}{kT}\right),$$

where *N* is the actual number of cycles.

Equation (6) forms the premise of establishing the fatigue damage evolution law; however, no theory can directly prove it. Therefore, it is necessary to deduce the specific form *Q*(*σ_a_*, *σ_m_*, *D*) from actual behavior characteristics of the fatigue damage process (damage and evolution rate, etc.).

#### 2.1.2. Damage Evolution Law under Single Cyclic Load and Constant Temperature

When the temperature and the load are constant, *Q*(*σ_a_*, *σ_m_*, *D*) is only a function of the damage variable *D*, and Equation (6) can be expressed as:

(7) $$\frac{dD}{dN}=A\exp\left(-F\left(D\right)\right).$$

When a material is damaged and fails, the damage variable *D* = 1 and critical activity energy *Q* = 0; thus, *F*(1) = 0. In other words, when $D_{C}\neq 1$, *D* can be understood as *D_i_*/*D_C_* (*D_i_* is the damage value, *D_C_* is the critical damage threshold of the material under fatigue failure). Once the theoretical expression for *F*(*D*) can be found, the material damage evolution law can be expressed well.

**Figure 1:**
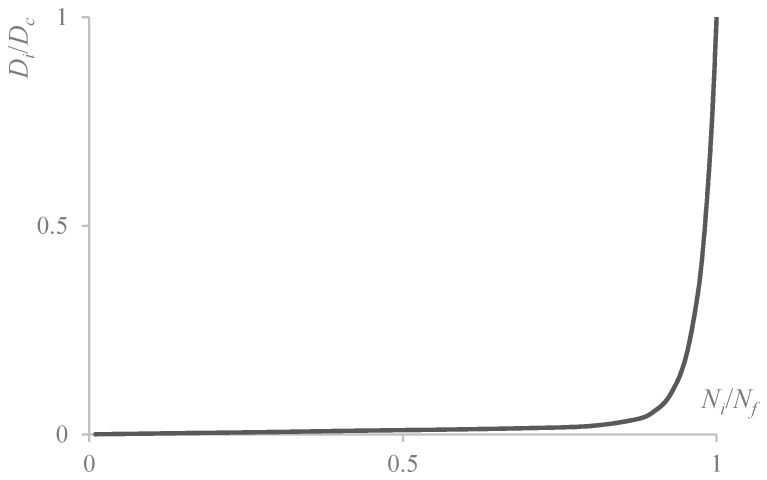
Relationship between damage variables and number of cycles.

Under a certain level of repeated load stress, the relationship between material damage variables and the number of repeated cycles can be generally qualitative, as shown in Figure 1 [39]. In Figure 1, *N_f_* is the number of cycles when fatigue failure occurs (which is related to the level of cyclic loads). The accumulation rate of damage generally increases with an increase in material damage. During the initial stage of fatigue loading, the damage amount and evolution rate are small, whereas the amount of damaged material will increase rapidly when the material is close to failure.

According to the mathematical description of the curve in Figure 1, Chaboche proposed an evolution law of fatigue damage [36], which can be expressed as:

(8) $$D=ax+b\exp(-c(1-x^{m})),$$

or

(9) $$x={\left(1+\frac{1}{c}\ln\left(\exp\left(-c\right)\right)+D\right)}^{\frac{1}{m}}.$$

In Equation (8), the first term on the right side is the linear part, corresponding to the linear cumulative damage, and *a* is the evolution of the rate of damage. The second term is the nonlinear part, corresponding to the mutual influence of damages after a certain extent of damage accumulation. Assume:

(10) $$x=N_{i}/N_{f},\;D=D_{i}/D_{C},$$

where *N_i_* is the actual number of cycles.

When *x* = *N_i_*/*N_f_* = 1 and *D* = *D_i_*/*D_C_* = 1, *b* = 1 − *a* is not an independent constant. Moreover, *c* and *m* are the material characteristic parameters. When *x* = 0, $D_{0}=\exp(-c)$ is the initial damage of the material. The damage process of materials can be well described by adjusting the parameters *a*, *c*, and *m*.

According to Equations (7) and (8):

(11) $$F\left(D\right)=-\ln\left[\left(a+\left(D-ax\right)\cdot cm\cdot x^{m-1}\right)\cdot A^{-1}\right].$$

From Equation (9), the damage evolution rate is:

(12) $$\frac{dD}{dN}=cm\cdot \left[D-a\cdot {\left(1+\ln\left(D+\exp\left(-c\right)\right)\cdot c^{-1}\right)}^{\frac{1}{m}}\right]\cdot {\left[1+\ln\left(D+\exp\left(-c\right)\right)\cdot c^{-1}\right]}^{\frac{m-1}{m}}+a.$$

When *F*(1) = 0, Equation (11) becomes:

(13) A=(1−a)cm+a.

If $f=a+cm\cdot \left[D-a\cdot {\left(1+\ln\left(D+\exp\left(-c\right)\right)\cdot c^{-1}\right)}^{\frac{1}{m}}\right]\cdot {\left[1+\ln\left(D+\exp\left(-c\right)\right)\cdot c^{-1}\right]}^{\frac{m-1}{m}}$, then

(14) F(D)=−ln(f/A).

When the value of *a* is small, the first term in Equation (12) only comes into play when the value of *D* is large:

(15) $$\frac{dD}{dN}=a+cm\cdot {\left(1+\ln\left(D+\exp\left(-c\right)\right)\cdot c^{-1}\right)}^{\frac{m-1}{m}}\cdot D.$$

The relation between fatigue life and damage is:

(16) $$N=\int_{D_{0}}^{D_{n}}\frac{dD}{M\left(D\right)},$$

where *D*_0_ is the initial damage under cyclic load, *D_n_* is the final damage after the secondary cycle, and *M*(*D*) is the right side of Equations (12) and (15).

If

(17) $$D=\frac{D_{n}-D_{0}}{2}\xi +\frac{D_{n}+D_{0}}{2}\left[35\right],$$

then Equation (16) becomes

(18) $$N=\frac{D_{n}-D_{0}}{2}\int_{-1}^{1}\frac{d\xi }{M\left(\frac{D_{n}-D_{0}}{2}\xi +\frac{D_{n}+D_{0}}{2}\right)}.$$

Owing to the complex form of *M*(*D*), when the value of *D_n_* − *D*_0_ is large, Equation (18) directly carries out Gaussian numerical integration with a large error. The piecewise integration form is adopted:

(19) $$N=\sum_{i=1}^{k}\Delta N_{i}=\sum_{i}\int_{D_{i0}}^{D_{in}}\frac{dD}{M\left(D\right)},$$

where *k* is the number of sections, $D_{i0}=\left(i-1\right)\left(D_{n}-D_{0}\right)/k$, and $D_{in}=i\left(D_{n}-D_{0}\right)/k$.

According to Equation (15), the dimensional damage evolution law can be expressed as:

(20) $$\frac{N_{f}}{D_{C}}\frac{dD}{dN}=a+cm\frac{D}{D_{C}}{\left(1+\frac{1}{c}\ln\left(\exp\left(-c\right)+D\right)\right)}^{\frac{m-1}{m}}.$$

*N_f_*, *D_C_*, and *a* are all related to the levels of cyclic loads. The dimensional damage evolution law is given as:

(21) $$\frac{dD}{dN}=\frac{D_{C\sigma }}{N_{f\sigma }}a+cm\frac{D}{N_{f\sigma }}{\left(1+\frac{1}{c}\ln\left(\exp\left(-c\right)+\frac{D}{D_{C\sigma }}\right)\right)}^{\frac{m-1}{m}}=a^{\prime }+\eta D{\left(1+\frac{1}{c}\ln\left(\exp\left(-c\right)+\frac{D}{D_{C\sigma }}\right)\right)}^{\frac{m-1}{m}},$$

where *a*, *N_f__σ_*, and *D_C__σ_* are related to the amplitude of the cyclic load (i.e., stress cyclic level *σ*), and *c* and *m* are material characteristics.

When the stress amplitude is lower than the fatigue limit *σ*_−1_, $N_{f\sigma }\to \infty$, and *N_f__σ_* can be obtained from Equation (21). For a single type of cyclic load stress, the integral of Equation (21) can be obtained as:

(22) $$N_{f\sigma }=\int_{0}^{D_{C\sigma }}dD/\left(\frac{D_{C\sigma }}{N_{f\sigma }}a+cm\frac{D}{N_{f\sigma }}{\left(1+\frac{1}{c}\ln\left(\exp\left(-c\right)+\frac{D}{D_{C\sigma }}\right)\right)}^{\frac{m-1}{m}}\right).$$

*a*, *D_C__σ_*, *c*, and *m* satisfy the following condition:

(23) $$\int_{0}^{D_{C\sigma }}\frac{dD}{\frac{D_{C\sigma }}{N_{f\sigma }}a+cm\frac{D}{N_{f\sigma }}{\left(1+\frac{1}{c}\ln\left(\exp\left(-c\right)+\frac{D}{D_{C\sigma }}\right)\right)}^{\frac{m-1}{m}}}=1.$$

Using $\xi =\frac{D}{N_{C\sigma }}$, then

(24) $$\int_{0}^{1}\frac{d\xi }{\frac{D_{C\sigma }}{N_{f\sigma }}a+cm\xi {\left(1+\frac{1}{c}\ln\left(\exp\left(-c\right)+\xi \right)\right)}^{\frac{m-1}{m}}}=1.$$

According to Equation (24), the evolution rate of the initial material damage is independent of the stress action level of repeated loads but is related to the initial damage and material characteristics. In Equation (21), the dimensionless initial damage rate *a*′ is related to the level of the cyclic stress.

#### 2.1.3. Damage Evolution Law under Different Cyclic Stress Amplitudes

By substituting $\sigma_{a}^{n}N_{f\sigma }=C$ into Equation (21):

(25) $$\frac{dD}{dN}=\frac{D_{C\sigma }a\sigma_{a}^{n}}{C}+\frac{cm\sigma_{a}^{n}}{C}D\times {\left(1+\frac{1}{c}\ln\left(\exp\left(-c\right)+\frac{D}{D_{C\sigma }}\right)\right)}^{\frac{m-1}{m}}.$$

The fatigue failure condition under a symmetrical cyclic load is given as:

(26) $$\sigma_{\max eff}=\frac{\sigma_{a}}{1-D_{C\sigma }}=\sigma_{b},$$

where *σ_b_* is the tensile strength of the material, and

(27) $$D_{C\sigma }=1-\frac{\sigma_{a}}{\sigma_{b}}.$$

Then, the damage evolution law under the same cyclic stress amplitude is written as:

(28) $$\frac{dD}{dN}=H_{1}\left(1-\frac{\sigma_{a}}{\sigma_{b}}\right)\sigma_{a}^{n}+H_{2}\sigma_{a}^{n}D\times {\left(1+\frac{1}{c}\ln\left(\exp\left(-c\right)+\frac{D}{\left(1-\sigma_{a}/\sigma_{b}\right)}\right)\right)}^{\frac{m-1}{m}}.$$

The coefficients *H*_1_ = *a*/*C* and *H*_2_ = *cm*/*C* are correlated. The four material parameters *c*, *m*, *C*, and *n* will be fitted according to the results of the fatigue experiments. From Equations (6) and (28), the critical activity energy under symmetrical cyclic loading can be expressed as:

(29) $$Q\left(\sigma_{a},D\right)=-kT\ln\left(\psi \left(\sigma_{a},D\right)/A\right),.$$

(30) $$\psi \left(\sigma_{a},D\right)=H_{1}\left(1-\frac{\sigma_{a}}{\sigma_{b}}\right)\sigma_{a}^{n}+H_{2}\sigma_{a}^{n}D\times {\left(1+\frac{1}{c}\ln\left(\exp\left(-c\right)+\frac{D}{1-\sigma_{a}/\sigma_{b}}\right)\right)}^{\frac{m-1}{m}}$$

#### 2.1.4. Fatigue Damage Accumulation

The fatigue damage life of the structure is the accumulation of damage under different levels of cyclic loads; its evolution law can be obtained using Equation (28).

When the material damage variable *D* is small, the damage increment caused by different repeated loads can be considered to be independent of the current damage of the material. Therefore, the second term on the right side of Equation (28) can be ignored:

(31) $$\Delta D=H_{1}\left(1-\frac{\sigma_{ai}}{\sigma_{b}}\right)\sigma_{ai}^{n}N_{i}=a\left(1-\frac{\sigma_{ai}}{\sigma_{b}}\right)\frac{N_{i}}{N_{fi}},$$

where *N_i_* is the number of *σ_ai_* repeated cycles, and *N_fi_* is the number of *σ_ai_* failure cycles. Thus, under different cyclic loads, the total damage can be directly accumulated linearly if the amount of damage is small:

(32) $$D_{Total}=\sum \Delta D\propto \sum N_{i}/N_{fi}.$$

The Miner accumulation law is given as $D_{C}=D_{Total}$, $\sum N_{i}/N_{fi}=1$.

When the damage variable *D* is large, the second term on the right side of Equation (28) cannot be ignored and plays a major role. This means that the damage accumulation of materials is nonlinear and increments of fatigue damage caused by different load stress amplitudes are related to the order of the load stress actions. Under the *n*th repeated load, the stress amplitude is *σ_ai_*, and *N_i_*—the increment of material damage $\Delta D=D_{i}-D_{i-1}$ caused by the *i*th repeated load—is computed as:

(33) $$N_{i}=\int_{D_{i-1}}^{D_{i}}\frac{dD}{\psi \left(\sigma_{ai},D\right)}.$$

Equation (33) is a nonlinear integral equation, which can be solved by a numerical method in the order of material damage accumulation. When a load $D_{n}=\sum \Delta D_{i}<D_{C\sigma }$, the fatigue load can continue to be sustained; otherwise, fatigue failure or failure is considered to occur:

(34) $$\frac{N_{i}}{N_{fi}}=\int_{D_{i-1}}^{D_{i}}dD/\left[a\left(1-\frac{\sigma_{ai}}{\sigma_{b}}\right)+cmD\times {\left(1+\frac{1}{c}\ln\left(\exp\left(-c\right)+\frac{D}{1-\sigma_{ai}/\sigma_{b}}\right)\right)}^{\frac{m-1}{m}}\right].$$

According to Equation (34):

(35) $$\Delta D=D_{i}-D_{i-1}=f\left(D_{i-1},N_{i}/N_{fi},\sigma_{ai}\right).$$

Therefore, the form of the cumulative damage law is:

(36) $$D_{C}=\sum f\left(D_{i-1},N_{i}/N_{fi},\sigma_{ai}\right).$$

### 2.2. Experiment

#### 2.2.1. Raw Materials and Pavement Performances of Asphalt Concretes on Long-Span Steel Bridge Decks

Epoxy asphalt (EA) is a common paving material used in long-span steel bridge decks [4]. In order to better characterize the material properties of EA, modified matrix asphalt (SMA) and guss asphalt (GA) were compared and analyzed. The aggregate gradation and the optimum ratio of stone to oil for EA, SMA-10, SMA-13, GA-I, and GA-II concrete are shown in Table 1, according to the corresponding Chinese standard test methods of bitumen and bituminous mixtures for highway engineering (JTG E20-2011) [40].

The pavement performance, void fraction, dynamic stability, bending strength/strain, low-temperature anti-crack property, rutting resistance, and water stability of asphalt concretes were examined according to JTG E20-2011 [40].

Table 2 shows the measured void fraction, dynamic stability (60 °C and 70 °C), ultimate flexural strength/strain (−15 °C, 1 mm/min), indirect tensile strength (25 °C), indirect tensile strength after the freeze–thaw cycle (25 °C), linear contraction coefficient (15 °C to −15 °C), and water stability. The technical standard requirements are also provided in Table 2.

It can be seen from Table 2 that the temperature stability of the EA mixture was the best among all the samples. Moreover, the linear shrinkage coefficient of the EA mixture was the smallest, which is closer to the linear shrinkage coefficient of the steel plate. These indicate that the temperature sensitivity of the EA mixture is small, and its performance at high and low temperatures is more balanced than that of the other mixtures (SMA and GA). Besides, the ultimate bending strength/strain of the epoxy asphalt mixture meets the technical requirements set forth by the standard.

**Figure 2:**
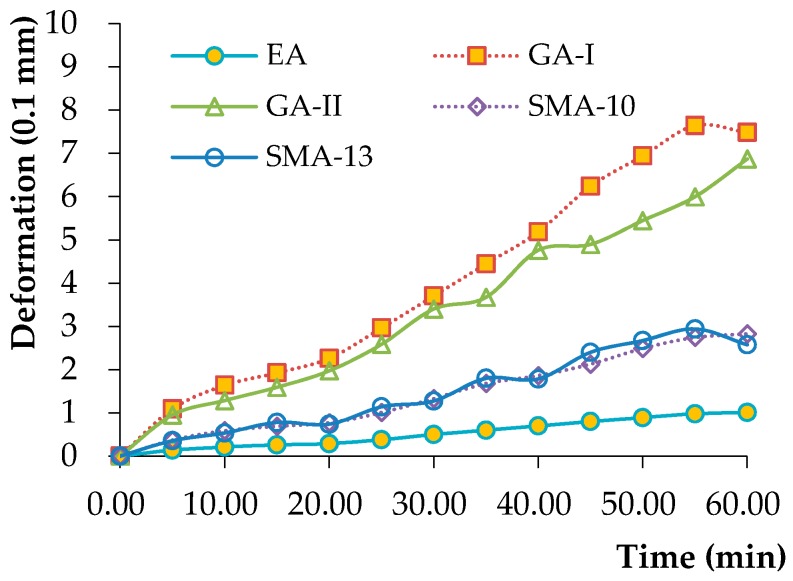
Rutting test results for asphalt concretes at 70 °C.

The thermal stability of the EA concrete was assessed by the rutting test (T0719-2011) [40]. The size of the sample used in the rutting test was 300 mm × 300 mm × 50 mm. The test was conducted at 70 °C under drying conditions since the highest temperature of the paving layer can reach 70 °C. During the rutting test, the pressure under the solid-rubber wheel was 0.7 MPa, and the traveling speed of the wheel was 42 cycles/min. The effects of temperature and stress level on the rutting performance of matrix asphalt concrete were very significant. With the increase of time, the amount of rutting deformation increases. The higher the temperature and load level were, the larger the rutting deformation increment was. Figure 2 shows the rutting deformation of the specimen over time. As far as deformation is concerned, GA was the largest, followed by SMA, with EA being the smallest. It is seen that the rutting deformation of the EA concrete was low; i.e., the rutting deformation at 60 min was only 1.01 mm, which indicates that the EA concrete used in this study possesses good stability at high temperatures.

**Figure 3:**
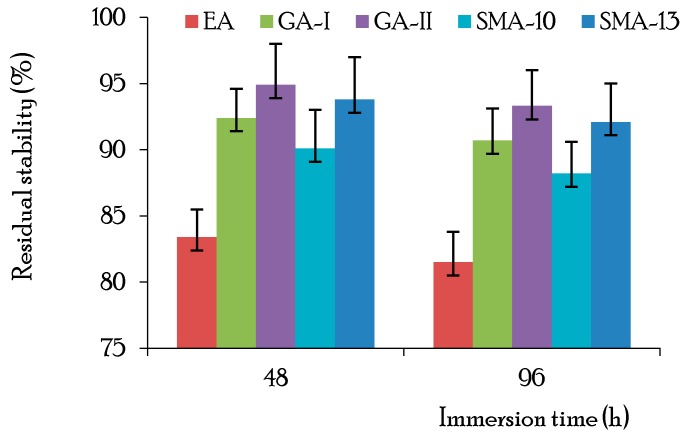
Immersion Marshall stability results for asphalt concretes.

The water stability of the asphalt concrete was evaluated by the immersion Marshall stability test (T0717-1993) [40]. The immersion Marshall stability test is similar to the standard test except that the specimens are immersed in water at constant temperature for 48 and 96 h. The measured residual stabilities of the immersed specimens are shown in Figure 3. It is seen that the average residual stability of the immersed specimens was greater than the design criterion (80%), and the immersion time had a minor effect on the residual stability, which indicates that all three types of materials used in this study possess good water stability [40].

#### 2.2.2. Fatigue Measurement of Asphalt Concrete Beam on Steel Plate

**Figure 4:**
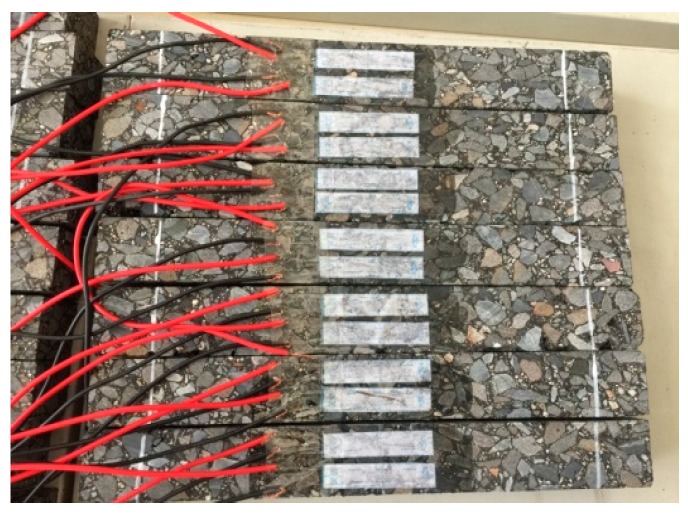
Test beam.

The Strategic Highway Research Program (SHRP) report provides a comprehensive evaluation and ranking of the degree of field simulations of different fatigue performance tests, feasibility of each of the test methods, feasibilities of the test results, and correlations with the test results. It is considered that the cyclic bending test can represent the actual stress state of asphalt pavements adequately, and the results can be directly applied to engineering design. Therefore, this paper uses the bending fatigue test for beams to evaluate the fatigue damage performance of the asphalt pavement. For each asphalt mixture, board specimens of dimensions 380 mm × 100 mm × 50 mm were formed according to the optimal asphalt ratio, and the compactness of the rut board was controlled at 98% of the Marshall compactness. Three beams from each group were tested simultaneously, as shown in Figure 4.

**Figure 5:**
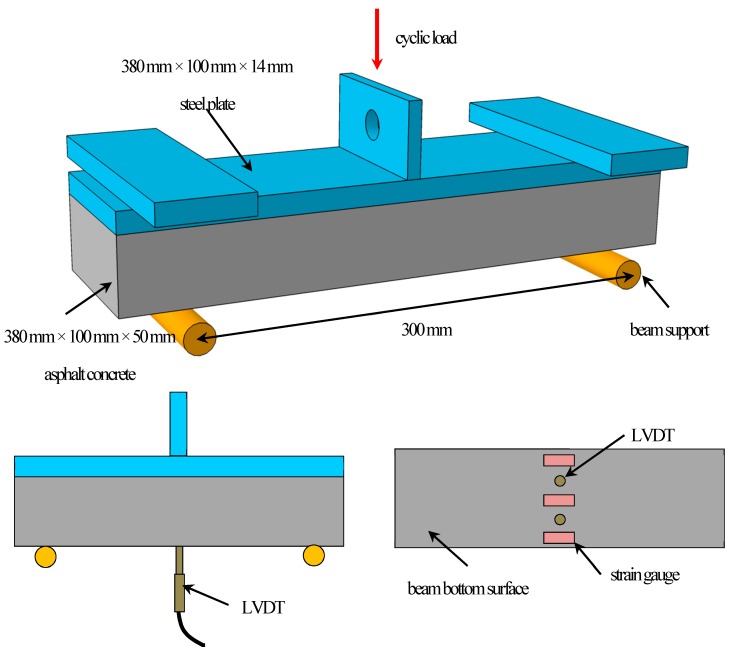
Schematic illustration of the fatigue test for asphalt concrete beam. (LVDT: linear variable differential transducer.).

The fatigue tests of the composite beams of asphalt concrete and steel plate were conducted on an MTS810 servo hydraulic material testing machine (MTS Industrial Systems (China) Co., Ltd., Nanjing, China). The schematic illustration of the fatigue test is shown in Figure 5. The testing equipment consisted of three parts: test facility, environment room, and data control and acquisition system.

Test conditions for the temperature were 15, 20, and 60 °C, and the test specimens were retained for more than 4 h under the given temperature condition ±0.5 °C. The fatigue load levels can be deduced from the equivalent stress method, and they were calculated to be 12 kN and 16 kN by considering the action of standard axle loads of BZZ-100kN and BJ-130kN, respectively [41]. The continuous partial sine loading mode controlled by constant stress (12 and 16 kN) was adopted here, and the loading frequency was 10 ± 0.1 Hz. Under the target load level, when longitudinal cracks were formed on the surface of the specimen, it was considered that the specimen was damaged, and the loading test on the specimen was stopped.

For the fatigue tests, three strain gauges were attached at the bottom of the midspan of the composite beam to measure the strain, and the average of the three measured strains was reported. Besides, the beam deflection at the midspan was measured by two linear variable differential transducers (LVDTs) during the fatigue test.

## 3. Results and Discussion

### 3.1. Fatigue Damage Evolution Law and Accumulation of Asphalt Concrete

During the fatigue test, the composite beam is considered to reach failure when one of the following conditions occurs: (1) cracks appear at the surface of the asphalt concrete; (2) cracking or slipping occurs at the bonding layer; or (3) lamination occurs in the asphalt concrete.

**Figure 6:**
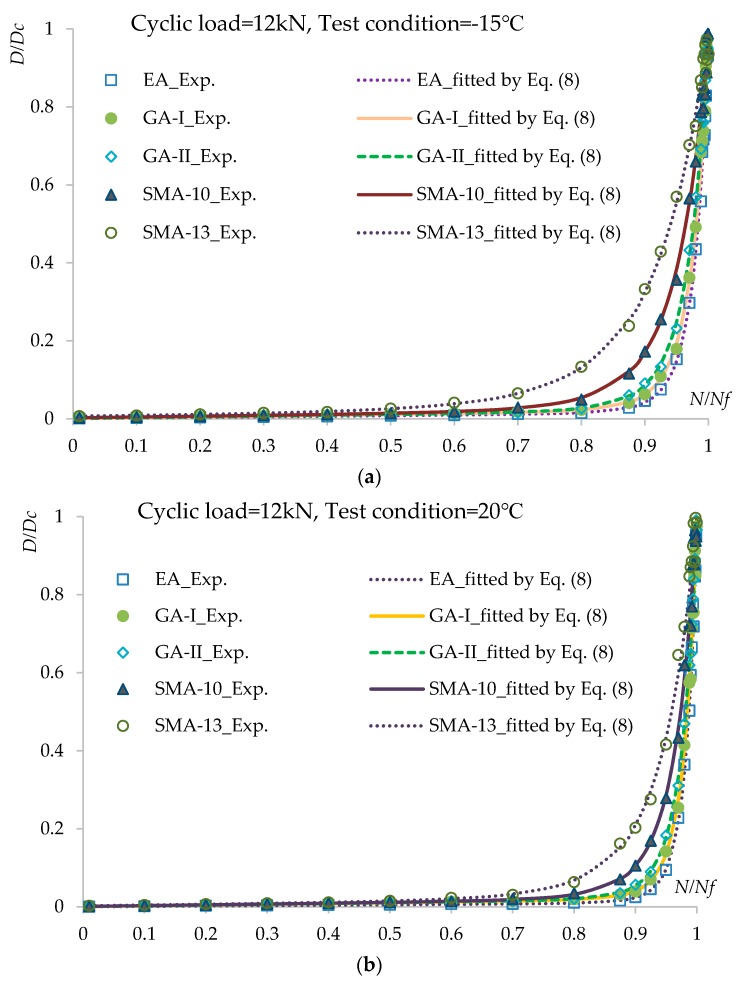

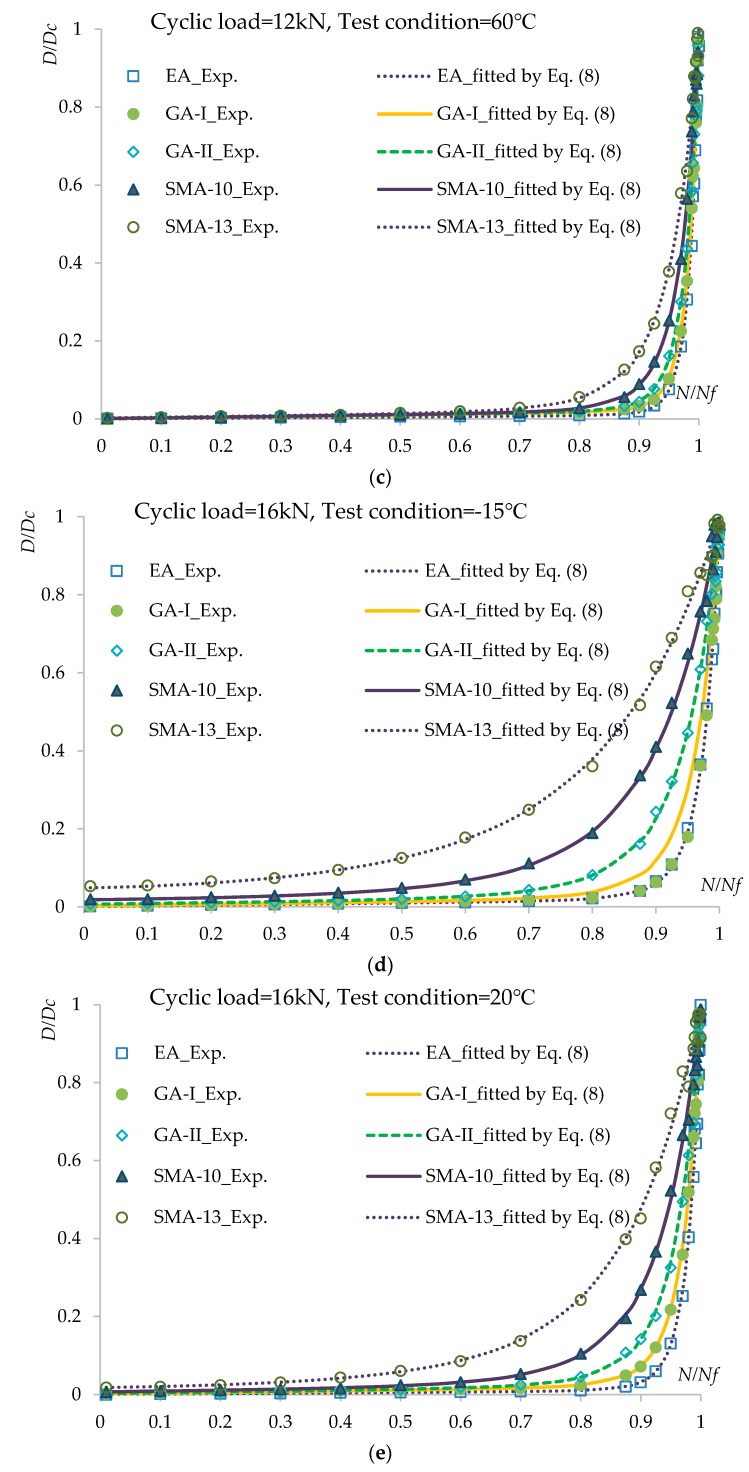

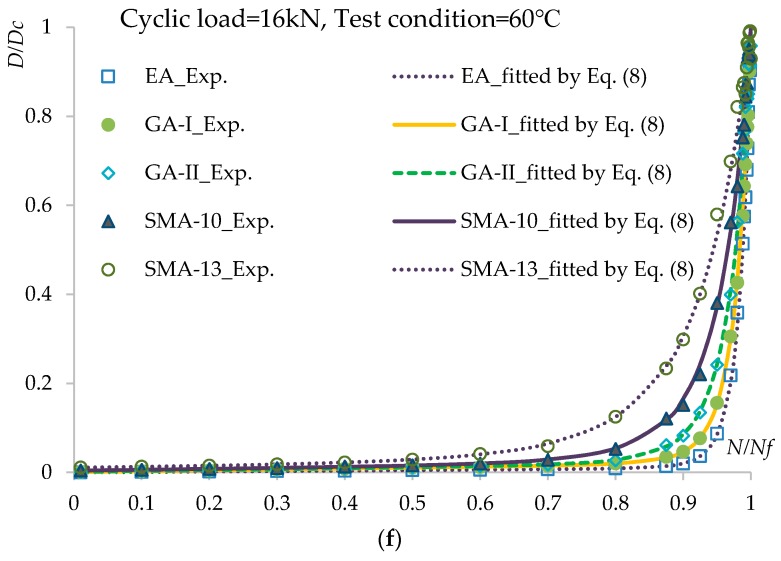
Relationship between *x* = *N*/*N_f_* and *D* = *D_i_*/*D_C_* under different loading cycle levels and test conditions: (**a**) cyclic load = 12 kN, test condition = −15 °C; (**b**) cyclic load = 12 kN, test condition = 20 °C; (**c**) cyclic load = 12 kN, test condition = 60 °C; (**d**) cyclic load = 16 kN, test condition = −15 °C; (**e**) cyclic load = 16 kN, test condition = 20 °C; (**f**) cyclic load = 16 kN, test condition = 60 °C.

In order to predict the fatigue lives of asphalt concretes under different loading levels by the damage-mechanics-based fatigue life model described in Section 2.1.2, one should determine the material parameters a, c, and m in Equation (8). The relationship between $x=N/N_{f}$ and $D=D_{i}/D_{C}$ under loading cycle levels of 10 and 12 kN and test conditions of −15, −20, and 60 °C for the fatigue tests are shown in Figure 6a–f. The results fitted by Equation (8) are also displayed in Figure 6 as curves. The fitted results are in good agreement with the experimental results, which suggests that Equation (8) can characterize the relationship between $x=N/N_{f}$ and $D=D_{i}/D_{C}$ well.

**Figure 7:**
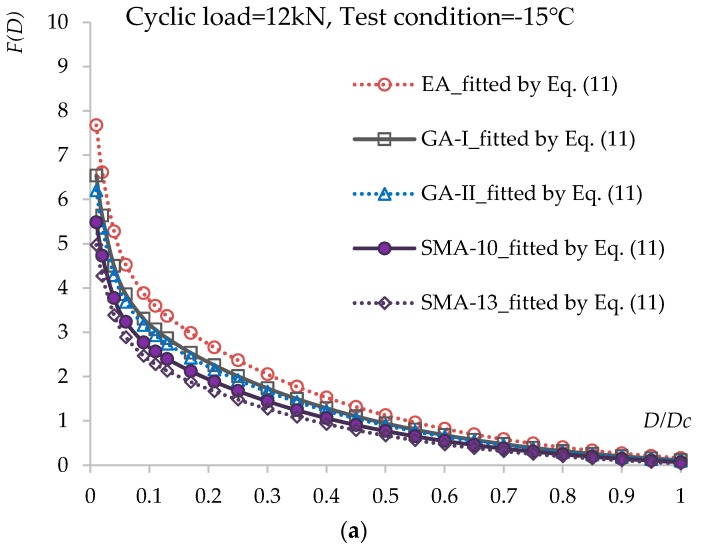

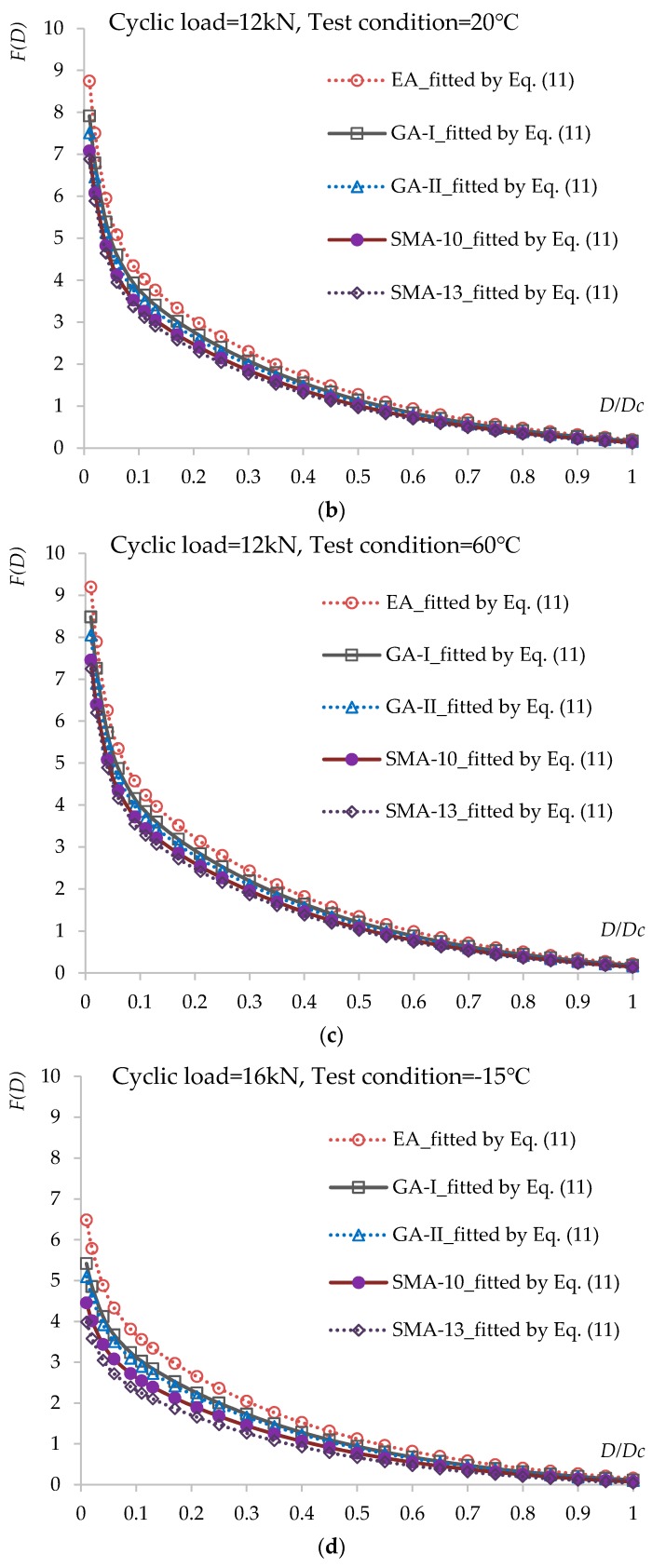

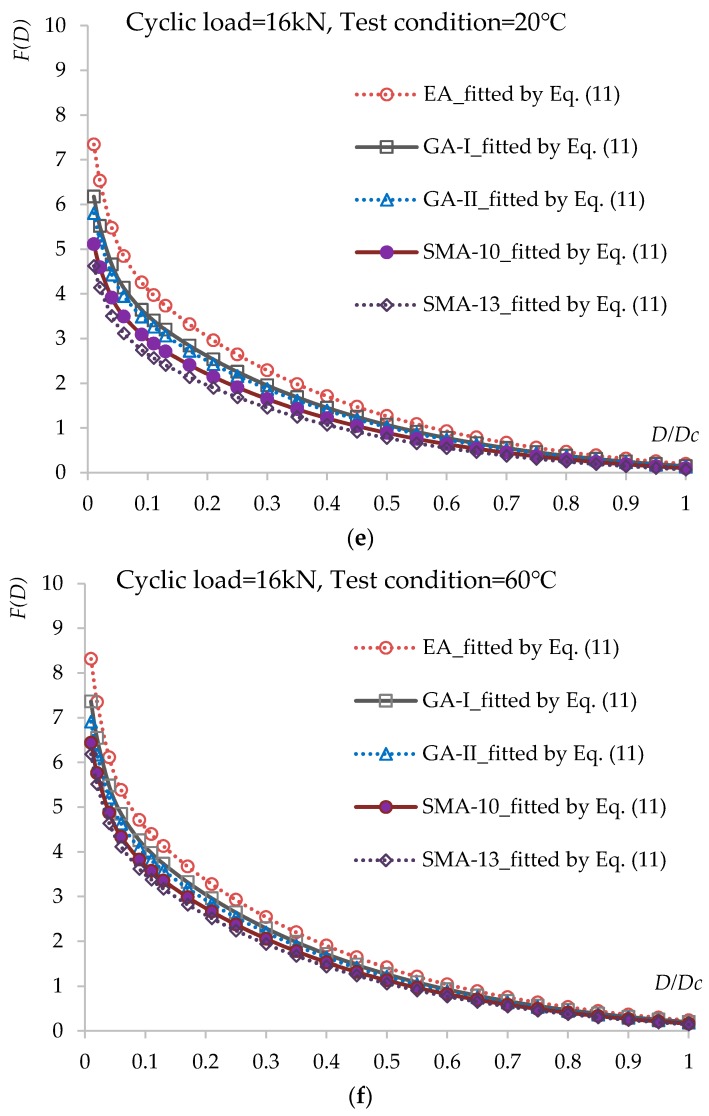
Relationship between accumulated energy *F*(*D*) and damage evolution *D* = *D_i_*/*D_C_* under different loading cycle levels and test conditions: (**a**) cyclic load = 12 kN, test condition = −15 °C; (**b**) cyclic load = 12 kN, test condition = 20 °C; (**c**) cyclic load = 12 kN, test condition = 60 °C; (**d**) cyclic load = 16 kN, test condition = −15 °C; (**e**) cyclic load = 16 kN, test condition = 20 °C; (**f**) cyclic load = 16 kN, test condition = 60 °C.

The trends in the critical active energy change caused by damage evolution under loading cycle levels of 12 and 16 kN and test conditions of −15, 20, and 60 °C for the fatigue tests are shown in Figure 7a–f. The results fitted by Equation (11) are also displayed in Figure 7 as curves. The fitted results are in good agreement with the experimental results, which suggests that Equation (11) can characterize the relationship between accumulated energy *F*(*D*) and damage evolution *D* = *D_i_*/*D_C_* well.

From the calculated results in Figure 6 and Figure 7, it is observed that the damage accumulation increases continuously under cyclic loads. The damage degree increases slowly during the early stage but speeds up in the middle and late stages. The higher the load level and the lower the temperature are, the greater the damage reduction is. By comparing among the asphalt concrete materials, it can be seen that for the rate of damage degree, EA, GA-I/II, and SMA-10/13 show increases in that order.

### 3.2. Prediction of Fatigue Life of the Asphalt Concrete on the Long-Span Steel Bridge

**Figure 8:**
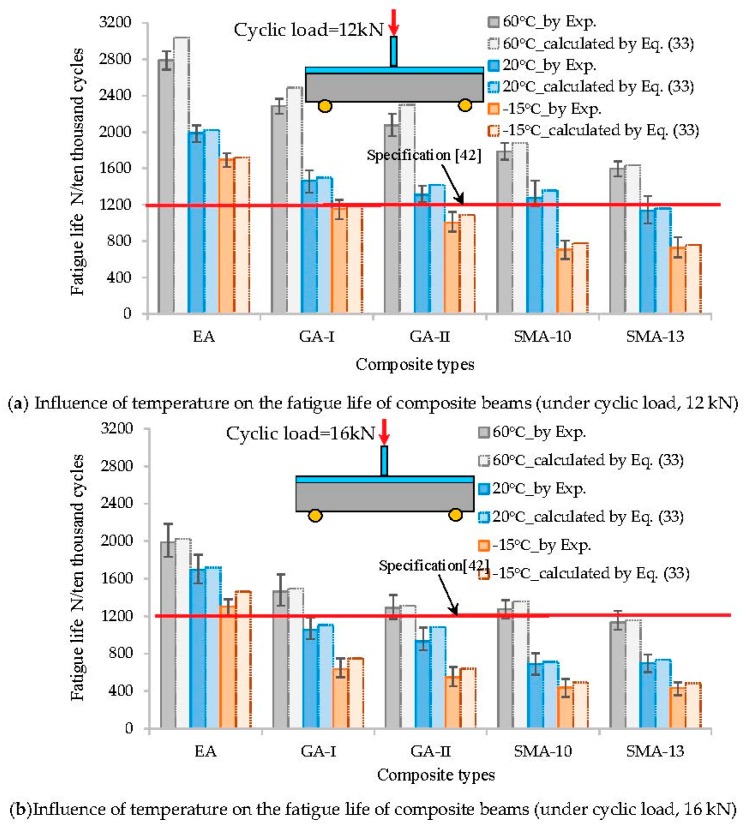
Comparison between fatigue life prediction and measurement under different loading cycle levels and test conditions (unit: ten thousand cycles): (**a**) cyclic load = 12 kN; (**b**) cyclic load = 16 kN.

By substituting the material parameters into Equation (33), the theoretical fatigue life expectancy of the bridge deck pavement can be obtained for different loading levels. The calculated results are shown in Figure 8.

According to the results in Figure 8,

1. The theoretical prediction of fatigue life is comparable to that from the bending fatigue test. The difference between them is not greater than 11%. However, the predicted theoretical value is larger than the experimental value. The reason for this is that the theoretical predicted value calculation assumes the material to be homogeneous and does not take into account the amplification effect of the initial defects such as pores and micro-cracks in the asphalt mixture on fatigue damage.
2. Temperature is one of the main factors affecting the fatigue life of asphalt pavement on a steel bridge deck. The fatigue life of the deck pavement decreases with temperature, as shown in Figure 8. This could be attributed to the increase in the elastic modulus of the asphalt mixture at lower temperatures, which results in an evident increase in the stress concentration and peak stress. Under the low-temperature condition of −15 °C, the EA deck pavement system achieved better anti-fatigue performance and could meet the design requirements of the steel deck pavement (*N_f_* > 1200, for 10,000 cycles) [42]. Moreover, EA mixtures have been successfully applied in the Third Nanjing Yangtze River Bridge (2005), the North Branch Bridge of the Runyang Yangtze River (2005), and the Sutong Yangtze River Bridge (2008), which have withstood the local climate and traffic conditions for over 10 years. However, GA-I/II and SMA-10/13 showed poor fatigue endurance at lower temperatures (*N_f_* < 12, for 10,000 cycles); thus, they failed to meet the design requirement for steel bridge deck pavement.
3. The effect of load levels on the fatigue resistance of the steel deck pavement is significant, as can be inferred from both the theoretical prediction and the experimental results under different load levels (Figure 8). The fatigue life noticeably decreased with the increase in load levels. Under the loading level of 12 kN, the fatigue cycle number (*N_f_*) was reduced by 15–20% compared with that of the load level of 16 kN. Additionally, the GA-I/II and SMA-10/13 showed poor fatigue endurance under overloading of 12 kN (*N_f_* < 12, for 10,000 cycles); thus, they failed to meet the design requirement for steel bridge deck pavement.
4. The bridge pavement constructed of EA has anti-fatigue performance superior to that of the other pavements. Under the same total thickness of the pavement layers, the fatigue life can be extended by a factor of more than one or two when GA-I/II or SMA-10/13 is adopted. The main reason is that the bending strength of the EA concrete is much higher than that of the modified SMA concrete and GA concrete. With higher strength and excellent resistance to deformation, the EA concrete is a suitable paving material for long-span steel bridge decks.
5. In fact, GA pavement has been widely used for deck pavement construction of long-span steel bridges in China, such as the Jiangyin Yangtze River Highway Bridge and the Tsingma Bridge in Hong Kong. Based on field applications, it is concluded that the pavements constructed of SMA show high rutting resistance and good adhesion of the interfaces; they are capable of withstanding the entire stress and meeting the basic requirements with respect to vehicle loads. However, their ability to withstand high temperatures is somewhat poor. To meet the requirement for high-cycle fatigue life, the total thickness of the pavement layers should be increased, which contradicts the design requirement for thin-layer steel bridge deck pavement.

## 4. Conclusions

In this paper, a fatigue life prediction model for the layered asphalt concrete pavement structure on a long-span steel bridge deck is established based on the mechanism of fatigue damage evolution of materials from the microscopic perspective, and the feasibility of the model is verified by fatigue tests. The following findings are obtained:

The proposed model can provide a reference to predict the fatigue life of the steel–asphalt concrete composite deck with cracked or non-cracked asphalt concrete layer. By fitting the material characteristic parameters of the damage evolution law through the splitting fatigue tests, the damage law can better simulate the change in the course of damage of different asphalt mixtures.

With higher strength and higher resistance to deformation, the EA concrete can provide better anti-fatigue performance under different loading conditions than the SMA and GA concretes. Therefore, the fatigue performance of bridge deck pavements can be considerably improved by using epoxy asphalt concrete as the pavement material

However, it is noted that the influences of environmental factors, processes of frost degradation of concrete, penetration of chlorides from the ice melting agents and transversal loading distribution coefficient on the fatigue life of the orthotropic steel–asphalt concrete composite deck are not considered in this study. In order to further improve the accuracy of the fatigue life models, the effects of the above factors should be incorporated into the prediction model of the steel–asphalt concrete composite deck in the future.

## Figures and Tables

**Table 1:** Design of mixture proportions in the experiment.

Type	Percentage of Particle Mass Passing through Different Sieve Holes (Square Sieve/mm) (%)										Asphalt Content (%)
	16	13.2	9.5	4.75	2.36	1.18	0.6	0.3	0.15	0.075	
EA		100	95–100	65–85	50–70	-	28–40	-	-	7–14	6.5
GA-I	-	100	90–100	60–80	50–70	-	35–40	28–35	25–34	20–27	7.2
GA-II	100	95–100	-	65–85	45–65	-	30–35	22–32	20–28	18–23	6.8
SMA-10	-	100	90–100	30–50	22–30	17–25	15–22	12–20	10–18	9–12	6.2
SMA-13	100	95–100	<80	22–36	20–30	16–26	14–22	12–19	10–17	8–12	6.0

**Table 2:** Pavement performance of asphalt concretes.

Performance	Test Condition and Unit	EA	GA-I	GA-II	SMA-10	SMA-13	Test Method	Requirement [40]
Void fraction	(%)	2.2	1.2	1.1	3	3.5	T0708-2011	≤1.5
Voids in the Mineral Aggregate	(%)	18.7	19.1	18.7	17.7	17.2	T0708-2011	>17
Asphalt filling rate	(%)	82.3	86.7	85.3	81.3	79.7	T0708-2011	70–90
Ultimate bending strength	−15 °C, 1 mm/min (MPa)	18.3	14.21	13.56	6.1	5.3	T0715-2011	≥10
Ultimate bending strain	−15 °C, 1 mm/min (10^−3^)	2.74	3.63	3.42	2.76	2.07	T0715-2011	≥2
Indirect tensile strength	25 °C (MPa)	5.39	4.05	3.95	1.65	1.37	T0716-2011	-
Indirect tensile strength after freeze–thaw cycle	(MPa)	4.15	3.77	3.09	1.41	1.24	T0729-2000	-
Linear contraction coefficient	15 °C to −15 °C (10^−5^ °C^−1^)	1.52	2.04	1.82	2.25	2.14	T0720-1993	≤3.00
